# Nerve Tracing in Juvenile Rats: A Feasible Model for the Study of Brachial Plexus Birth Palsy and Cocontractions?

**DOI:** 10.1055/s-0044-1778691

**Published:** 2024-01-22

**Authors:** Krister Jönsson, Tomas Hultgren, Mårten Risling, Mattias K. Sköld

**Affiliations:** 1 Department of Handsurgery Södersjukhuset, Karolinska Institutet Department of Clinical Science and Education, Södersjukhuset, Stockholm, Sweden; 2 Department of Neuroscience Karolinska Institutet, Experimental Traumatology Unit, Sweden; 3 Department of Medical Sciences, Section of Neurosurgery, Uppsala University, Sweden

**Keywords:** brachial plexus birth injury, cocontraction, Fluoro-Gold, rat

## Abstract

Brachial plexus birth injuries cause diminished motor function in the upper extremity. The most common sequel is internal rotation contracture. A number of these patients also suffer from cocontractions, preventing the use of an otherwise good passive range of motion in the shoulder. One theory behind the co-contracture problem is that injured nerve fibers grow into distal support tissue not corresponding to the proximal support tissue, resulting in reinnervation of the wrong muscle groups. To further elucidate this hypothesis, we used rat neonates to investigate a possible model for the study of cocontractions in brachial plexus birth injuries. Five-day-old rats were subjected to a crush injury to the C5–C6 roots. After a healing period of 4 weeks, the infraspinatus muscle was injected with Fluoro-Gold. A week later, the animals were perfused and spinal cords harvested and sectioned. Differences in the uptake of Fluoro-Gold and NeuN positive cells of between sides of the spinal cord were recorded. We found a larger amount of Fluoro-Gold positive cells on the uninjured side, while the injured side had positive cells dispersed over a longer area in the craniocaudal direction. Our findings indicate that the method can be used to trace Fluoro-Gold from muscle through a neuroma. Our results also indicate that a neuroma in continuity somewhat prevents the correct connection from being established between the motor neuron pool in the spinal cord and target muscle and that some neurons succumb to a crushing injury. We also present future research ideas.

## Background

Brachial plexus birth palsy (BPBP) affects roughly 2.9/1,000 live births in developed countries. 1 The vast majority (over 70%) go on to recover full or near full function without any surgical intervention. 2 Children who do not recover or only recover partly most commonly suffer from an internal rotation contracture in the glenohumeral joint. The contracture keeps the forearm in a position close to the torso compromising the ability to place the hand in front of the body. Reaching the mouth, head, and neck can become difficult. 3 4 Around 45% of children with external rotation contracture also develop some degree of incongruence in the glenohumeral joint. 5

The internal rotation contracture was described in detail as early as 1905, 6 but despite many studies, clinical as well as experimental, the mechanism behind this dysfunction is not clear. Today it is well established that contracture develops in the subscapularis muscle and that a release of that musculotendinous unit is key to effective treatment. 7

We have performed more than 400 subscapularis elongations on children with internal rotation contracture in our unit, with good clinical results in the passive and active shoulder motion in most patients. 4 5

In follow-up after surgery, we have observed a number of patients who cannot fully use their available range of rotation, due to simultaneous activation of the external rotators and their antagonists—a phenomenon known as “co-contraction.” This can affect both those with milder C5–C6 injuries and patients with more severe injuries including avulsions. Little is known about this phenomenon; it is unclear why some patients are affected more than others and to what extent it contributes to the development of contracture in the first place. 8 9 Even less known is the exact neurological mechanism behind this phenomenon.

Cocontractions about the shoulder have been investigated earlier in research concerning subacromial shoulder pain in adults, where it has been shown that cocontractions in the shoulder adductors, for example, latissimus dorsi during abduction, have been associated with less subacromial pain. 10 The cause of cocontractions in BPBP, however, is so little investigated that it has been stated as unknown as recently as 2019. 11 The idea of “misguided” or misled nerve healing has been suggested to be the cause of this but without conclusive evidence. 8 9 To the best of our knowledge, there has not been any experimental studies regarding the origins of the cocontractions seen in BPBP.

Rats and mice have a brachial plexus anatomy that is similar to that of humans and are therefore potentially useful as models for research on BPBP. When subjugated to a nerve injury on C5–C6 early in life, they have been shown to develop both external rotation contracture and the typical bony deformities of BPBP. 12 13 14

Fluoro-Gold (FG) is a well-known neural tracer. It can be injected directly into muscles, where it is taken up by the motor endplates and retrogradely transported into the central nervous system and the nerve cell body. 15

In this experimental study, our aim is to investigate a possible model for the study of cocontractions in BPBP and to elucidate if mechanisms such as cross-innervation 9 may have a role in causing cocontractions in BPBP by changing the motor neuron topography in the cervical cord. The null hypothesis was that there are no significant differences in FG uptake and distribution between injured and uninjured side.

## Materials and Methods

### Ethical Guidelines

The study was carried out according to ethical approval for animal experiments from the Stockholm board for ethics in animal experiments under number 9876-2018.

### Materials

Fourteen 5-day-old Sprague-Dawley rat pups from two different mothers were used for this study. They were operated in groups of five and nine. The rat pups all underwent the same procedure on the same side utilizing the uninjured side as control. Rats were kept together with their mother according to animal welfare institution guidelines. Water and food were provided ad libitum. All animals from each of the two litters were included. In the first operated group, two animals were lost during surgery due to blood loss and two more were euthanized due to signs of infection and ill being.

### Surgery

**Fig. 1:**
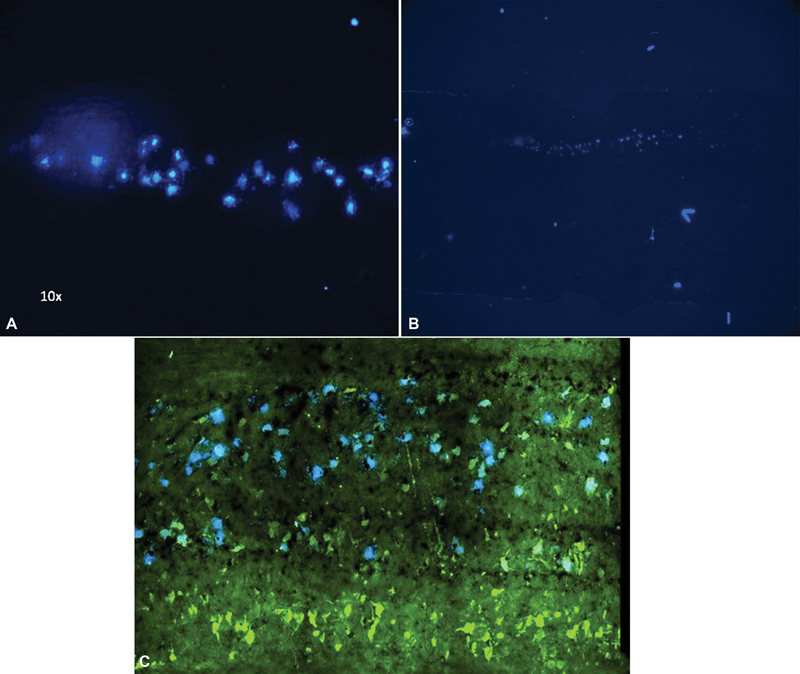
Specimens from the older test subjects when the method was developed. ( **A** ) Fluoro-Gold positive cells in coronal cut along the anterior horn on one side. ( **B** ) Coronal cut of the anterior horns of the spinal cord with unilateral uptake of Fluoro-Gold. ( **C** ) Coronal cut through both anterior horns demonstrating NeuN ( *green* ) on both sides and Fluoro-Gold ( *blue* ) on one side.

To determine the areas of interest for sectioning a prestudy using the protocol of surgery, injection and perfusion as described below were performed. The animals in the prestudy were thus treated the same as the animals in the two groups, but they were cut in the coronal plane to match the FG uptake more clearly to the cervical root level ( Fig. 1 ).

### Surgical Procedures

The 5-day-old pups were anesthetized using isoflurane, kept on a heat pad linked to a rodent rectal thermometer and thermostat, and the O _2_ saturation was monitored (all apparatus from Agnthos, Lidingö, Sweden). A surgical microscope 6-10x magnification (Leica, Germany) was used for dissection. In field sterility, a straight incision was made 1 mm below the clavicle, and the cephalic vein was heat coagulated and displaced cranially. The pectoralis and the sternomastoideus were split between fibers and held using micro-retractors. The suprascapular nerve was identified to find the upper trunk, and the upper trunk was then verified using a nerve stimulator (Bovie Neuro-pulse, Bovie Medical Corporation, United States) to elicit activity in abduction and elbow flection. To create a neuroma in continuity or axonotmesis, a constant pressure was held against the upper trunk proximal to the suprascapular nerve branching for 10 seconds using micro-forceps. 16 17 The fat pad was replaced, and the incision closed using interrupted Prolene 7–0 sutures. The rat pups were monitored in a separate clean and heated cage until showing signs of anesthesia wearing off, then returned to the mother. Perioperative fluid and peri- and postoperative analgesia were administered according to bodyweight via intraperitoneal injections of saline, Buprenorphine 0.05 mg/kg (Temgesic, Reckitt Benckiser Healthcare, UK), and Carprofen 5 mg/kg (Rimadyl Bovis vet, Zoetis, Finland).

Reduced forelimb function with pronation and elbow flexion was recorded in all subjects immediately postoperatively.

Four weeks postsurgery the rat pups were anesthetized again. In field sterility, the hair over the scapulae was shaved and the surgical loupes 3.5x magnification (Designs for vision, USA) were used for dissection. An incision was made over each scapula, exposing the underlying muscle but preserving the fascia to act as a seal around the injection needle. Into the angle between the body and the spina scapulae, that is, into the infraspinatus muscle, a Hamilton syringe was introduced. Ten microliters of FG 4% (Fluorochrome LLC, United States) was injected. The injection time was 1 minute per muscle to allow uniform distribution and prevent leakage. At the retraction of the needle, a cotton swab was used to prevent leakage of FG into the surrounding tissue. The wounds were closed using interrupted Prolene 7–0 sutures.

One week after the injections, the rats were deeply anesthetized. A perfusion needle was inserted into the left ventricle, the right atrium was opened, and the animals were perfused with 100-mL cold saline followed by 200-mL 4% [w/v] formaldehyde solution (Histolab, Sweden). The spine was dissected free from the paravertebral muscles and laminectomy was performed using micro-scissors. A micro-suture was attached cranial and posterior directly into the spinal cord for orientation. One group ( *n* = 9) was marked with a surgical marker at the C5–C6 level before dissecting it free from the spinal canal. The spinal cord was lifted during meticulous dissection from the spinal canal and post fixed in 4% [w/v] formaldehyde solution.

### Specimen Treatment

After 24 hours in 4% [w/v] formaldehyde, the specimens were cryoprotected in 20% sucrose solution. The specimens were frozen on dry ice, mounted, and transversally cut into 14-µm sections on a cryostat (Leica, Germany).

One group of specimens (Group I) ( *n* = 5) was cut from C1 and as caudal as the tissue was intact (21.5 mm), for investigations of the craniocaudal extension of FG uptake. The second group of specimens (Group II) ( *n* = 9) was sectioned through the injured C5–C6 level to provide information on differences between the sides. All sections were mounted on gelatin-coated slides and air dried.

From Group I, every fifth section was thawed for inspection of FG uptake. For Group II, every section was thawed and inspected for FG uptake.

Sixty sections from Group II (20 sections from three different animals) were stained with NeuN antibody 1:1,000 (Millipore Corporation, United States) by marking the edges of the slides with PAP PEN, re-moisturizing the specimens and applying NeuN antibody 1:1,000, phosphate buffer, and normal serum. The specimens were incubated in refrigerator for 18 hours, rinsed in phosphate buffer solution, and treated with donkey antimouse 1:400 before final rinse and covering.

The sections were examined under fluorescent microscope (Nikon Eclipse Ni-E, Nikon, Japan) and photographed using the attached camera (Andor Zyla sCMOS, Oxford Instruments, UK). For FG detection, the built-in Nikon-made DAPI filter (excitation 340–380 nm, emission 435–485 nm) was used. For NeuN detection, the bult-in Nikon-made FITC filter (excitation 465–495 nm, emission 515–555 nm) was used. The pictures were captured using NIS Elements 5.02 (Nikon). One animal specimen from group I was photographed in its entirety. These photographs were processed in Photoshop (CS5 Adobe, United States) and stitched together to a 3D model using Neurolucida (Neurolucida 360, MBF Bioscience, United States).

### Statistics

All data were recorded in SPSS version 28 (IBM, United States). The normality assumption of data was tested using the Shapiro–Wilk test. The Wilcoxon signed-rank test was then used to evaluate the differences between the injured and uninjured sides. A *p* ≤ 0.05 was considered significant.

## Results

**Fig. 2:**
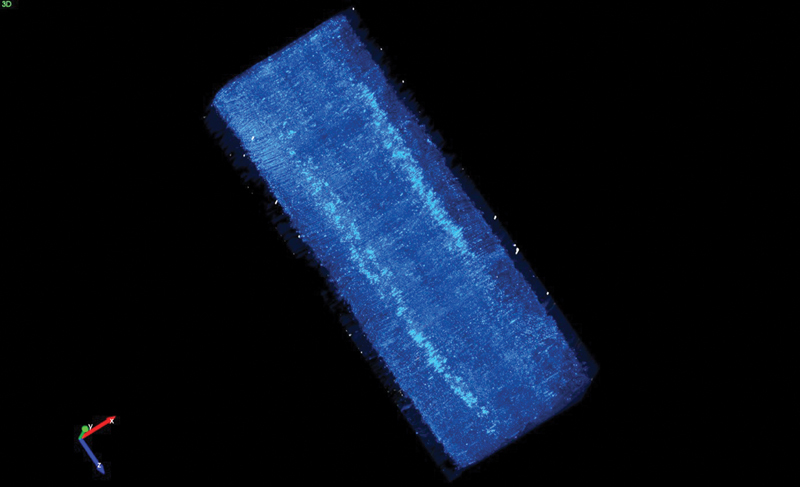
Visual representation of the dispersion of Fluoro-Gold (FG) positive cells on the respective sides. The image consists of 700 transverse images stacked together and rendered into a 3D structure. The wider dispersion and lower density of FG positive cells can be seen on the injured side ( *bottom left* ).

**Fig. 3:**
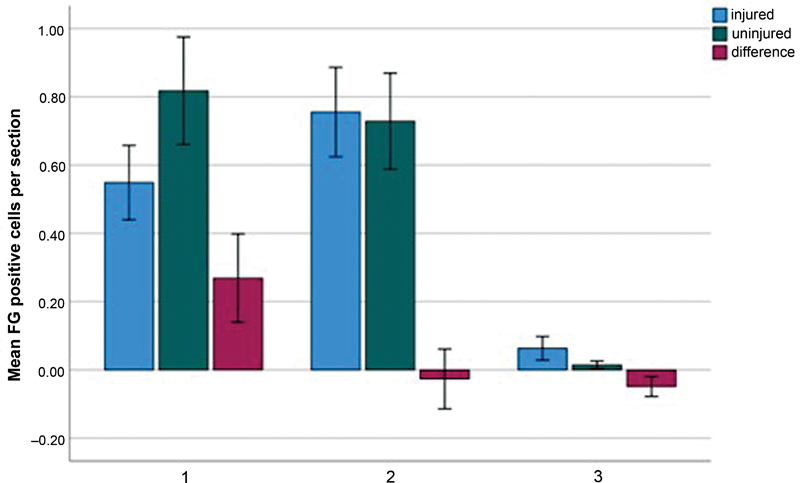
Bar chart showing average Fluoro-Gold (FG) positive cells at three equally distributed parts of the spinal cord cut along ∼21 mm; the parts are organized in thirds from cranial to caudal, with the first part containing the injured C5–C6 root; error bars represent the 95% confidence interval.

For Group I, where a long section of the spinal cord was harvested, we saw small differences between sides in total numbers but found a wider dispersion of the FG positive cells on the injured side ( Figs. 2 and 3 ).

On the injured side, we saw an average of 0.4594 (SD = 0.54617) positive cells for FG per section. For the uninjured side, there was on average 0.5230 (SD = 0.66407) positive cells for FG per section. The difference in FG positive cells per section of 0.0635 (SD = 0.44192) was not statistically significant ( *Z* = –1.578, *p* = 0.115).

When comparing the sides from the Group II where C5–C6 was selected and cut, we saw significant and more concentrated differences between sides.

**Fig. 4:**
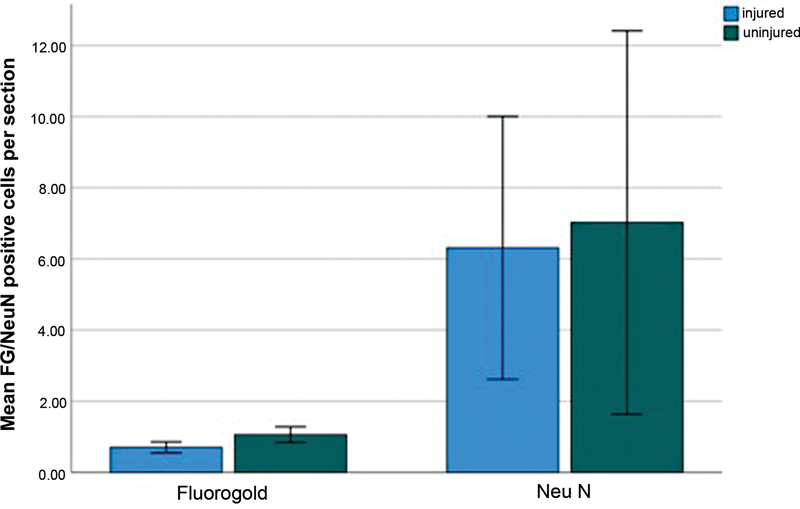
Bar chart showing average Fluoro-Gold and NeuN positive cells per section in group 2 at the C5–C6 level; error bars represent the 95% confidence interval.

On the injured side, we saw an average of 0.7073 (SD = 0.71961) positive cells for FG per section. For the uninjured side, there was an average of 1.0663 (SD = 1.0663) positive cells for FG per section. The difference in FG positive cells per section of 0.3590 (SD = 0.77790) was statistically significant ( *Z* = –3.912, *p* < 0.001; Fig. 4 ).

The average NeuN positive cells per section was 6.3123 (SD = 1.48605) and 7.0276 (SD = 2.16876) for the uninjured and injured sides, respectively. The difference in NeuN positive cells per section was not statistically significant ( *Z* = –1.069, *p* = 0.285; Fig. 4 ).

## Discussion

In this investigation, we have found differences in FG uptake between sides; however, statistical significance was only achieved for one group. For the other and the compiled material, no significant differences were found in the number of FG positive neurons. The null hypothesis cannot be rejected, albeit the method can be used to trace topographical reorganization in the spinal cord after nerve injury in continuity in rat neonates. This study is limited by the small number of animals, that the groups are not identical and that co contractions were not recorded. We note however that the standard deviation in FG uptake differs between sides, indicating a more dispersed innervation on the injured side. This is confirmed by ocular inspection of the specimens where the FG positive neurons on the injured side seem to be less densely packed together and dispersed over a longer area of the spinal cord ( Fig. 2 ). This indicates that the total amount of FG positive cells (here representing the number of neurons in contact with the muscle) is similar on both sides, but the neurons on the uninjured side are more concentrated or closely packed together, while those on the injured side are almost as numerous but spread thinly over a larger section of the spinal cord. There are some overlaps in the craniocaudal limits of motor neuron pools of different muscles in the common rat. 18 It is, however, reasonable to expect that the wide spread of the injured sides motor neuron pool would overlap with the anatomical motor pool location of other muscles in the anterior horn. The lower amount of NeuN positive cells on the injured side shows that some neurons succumb to the injury and the deprivation of input and neurotrophic factors during the healing process. 19 20 This was anticipated as a proportion of injured motor neurons as well as interneurons go through upregulation of apoptotic activity in rats injured at this age, 19 21 when deprived of afferent input, neurotrophic factors from contact with the periphery, or a combination thereof. The apoptotic upregulation can be partly inhibited by providing the neurons with neurotrophic factors. 20

The differences in absolute numbers between sides, although statistically significant, are small, and the number of specimens is limited. The groups of animals are not size-matched, and we interpret our results with great caution. We have, however, shown a trend toward a different anatomical dispersion after injury and that this is a viable method to study the regeneration of nerve paths in neurologically developing rat neonates, giving opportunity to further study not only BPBP but also other afflictions of the locomotor system in young individuals.

Similar investigations on nerve injuries in continuity on very young individuals have, to our knowledge, not been performed earlier. When looking for a model to investigate cocontractions in very young individuals sustaining nerve injury in continuity, we had no prior knowledge of the origin of the cocontraction.

Two main theories behind cocontractions can be postulated. It may be a central mechanism originating in the motor cortex that is disorganized. This would then presumably be due to lack of continuous contact with the periphery. Something similar to that seen in adult rats with nerve stem injuries, the normal architecture of the motor cortex is disrupted. 22 It may also be a peripheral mechanism originating from crisscross reinnervation inside a neuroma in continuity, for example, axons growing from neurons in the motor pool supplying the infraspinatus muscle ending up exerting motor control to the supraspinatus or the subscapularis muscle and therefore upon activation produce contractions in two or several antagonistic muscles at the same time.

The adult brain has enough plasticity to achieve some control over the sensation from a limb or a part of a limb with a severed nerve stem, 23 and children usually have better results after traumatic nerve injuries. 24 So why does this not apply to BPBP? In these injuries, results depend on the plasticity of the brain, that is, the ability to relearn a new pattern of activation. BPBP occurs at birth, causing a lack of input to a brain that is maturing very fast. We know that visual input is crucial during some development stages for visual cortex development. 25 Similarly a maturing motor cortex on the affected side in BPBP may not get the chance to develop the correct organization for activation.

Motor neurons depend for their survival on signals from their vast network of synapses and support tissue. In newborn animals, especially, an injury to the axon may compromise the survival of the neuron, while the mature neuron is more resilient to injury. 19 26 An injured motor neuron sets off a cascade of signaling and protein fabrication to change from the normal state to a producer of a new axon. Cell preservation proteins such as BDNF and axonal growth proteins like GAP-43 are produced in increasing quantities. 26 Cell adhesion proteins are upregulated by the proximal stumps' contact with the periphery; adhesion proteins, like the survivability after nerve injury, change depending on age at injury. 27

We interpret the wider dispersion on the injured side as FG being transported retrogradely through the axons, which at the point of the neuroma take a different path toward the spinal cord, much like a train going through a switch ending up at a different track. This might cause a cocontraction if several muscles were involved, and this was what Roth suggested following electrophysiological investigations. 9 However, we see the same phenomenon of faulty reinnervation in all nerve stem injuries. 23 These patients may experience transient cocontractions during rehabilitation, but, to the best of our knowledge, they do not persist in nerve stem injuries in children or adults. 24

The phenomenon of persisting cocontractions in BPBP and the reason for the difference in nerve stem injuries in older children merits further study, and we see multiple routes ahead. The presence and frequency of cocontractions in BPBP model rat neonates should be recorded using continuously surveilling electromyography. Any further animal model should also correlate the expression of cell regenerating proteins to the degree of function/contracture and the presence of cocontractions, and to the degree of dispersion outside the normal motor pool distribution. Further elucidation of the mechanisms behind cocontractions could help in developing specific treatments for this subgroup of patients, surgically and/or more targeted rehabilitation programs.
